# 

**Published:** 1905-12

**Authors:** 


### BOOKS RECEIVED.

Memoranda on Poisons. By Thomas Hawkes Tanner, M.D., F.L.S. Tenth revised edition by Henry Leffmann, A.M., M.D., Professor of Chemistry in the Woman's Medical College of Pennsylvania etc. Philadelphia: P. Blackston's Son & Co. 1905. (Price $. .75)

Mount Sinai Hospital Reports, vol 4, for 1903 and 1904. Edited for the Medical Board by N. E. Brill, A.M., M.D. Octavo, pp. 418. Stettiner Brothers, Printers, New York. 1905.

Operative Surgery. For Students and Practitioners. By John J. McGrath, M.D., Professor of Surgical Anatomy and Operative Surgery at the New York Post Graduate Medical School, Surgeon to the Harlem, Post Graduate, and Columbus Hospitals, New York. Second edition, thoroughly revised. With 265 illustrations, including many full-page plates in colors and half-tone. 628 Royal Octavo pages. (Extra cloth, $4.50 net; half morocco, $5.50 net.) Sold only by subscription. F. A. Davis Co., Publishers, 1914-16 Cherry st., Philadelphia, Pa.

Disorders of Metabolism and Nutrition. A series of Monographs. By Prof. Carl von Noorden,M.D., Physician-in-Chief to the City Hospital, Frankfort-on-Main. Authorised American edition. Translated under the direction of Boardman Reed,M.D. Part 7, Diabetes Mellitus: A Special Course of Lectures. Delivered in the University and Bellevue Hospital Medical College, New York. (Small 8vo, 212 pages. Price: $1.50) E. B. Treat & Co., Publishers, 241-3 West 23rd street, New York.

Neurotic Disorders of Childhood. Including a study of auto- and intestinal intoxications, chance anemia, fever, eclampsia, epelepsy, migordine, chorea, hysteria, asthma, etc. By B. K. Rachford, M.D., Professor of diseases of Children, Medical College of Ohio, Univer- and Jewish Hospitals. Octavo, pp. 440. New York: E. B. Treat & Co. 1905. (Price $2.75)

A Treatise on the Nervous Diseases of Children, for Physicians and Students. By B. Sachs, M.D., Alienist and Neurologist to Bellevue Hospital; Neurologist to the Mt. Sinai Hospital, consulting Physician to Manhattan State Hospital, east and west. Second edition. revised. Octavo. pp. 12—571. New York. William Wood & Co. (Price: $4.25)

A Textbook of Anatomy. Edited by D. J. Cunningham, F. R. S., M.D., (Edin. and Dubl.), D.Sc., LL.D. (Glasg. and St And.), D.C.L. (Oxford), Professor of Anatomy, University of Edinburg. Second and thoroughly revised edition. Illustrated with 936 wood engravings from original drawings, many in colors. Imperial Octavo, pp. 1388. New York: William Wood & Co. 1905. (Price: $6.00)

Biographic Clinics. Volume 3. Essays concerning the influence of visual function, pathologic and physiologic, upon the health of patients By George M. Gould, M.D., Editor of American Medicine, author of "An illustrated Dictionary of Medicine, Biology, etc. 12mo. pp. 516. Philadelphia: P. Blakiston's Son & Co. 1905. (Price $1.00)

International Clinics. A Quarterly of Illustrated Clinical Lectures and especially prepared articles on Treatment, Medicine, Surgery, Neurology, Pediatrics Obstetrics, Gynecology, Orthopedics, Pathology, Dermatology, Opthalmology, Otology, Rhinology, Laryngology, Hygiene, and other topics of interest to students and practitioners. By leading members of the medical profession throughout the world. Edited by A. O. J. Kelly, A.M., M.D., Philadelphia. Volume 3. Fifteen series. 1905. Philadelphia and London: J. B. Lippincott Co. (Cloth, $2.00)

Pathogenic microorganisms including Bacteria and Protozoa. A practical manual for students, physicians, and health officers. By William Hallock Park, M.D., Professor of bacteriology and hygiene, University and Bellevue Hospital Medical College, and director of the research laboratory of the New York Department of Health; assisted by Anna W. Williams M.D., assistant director of the research laboratory . Second edition, enlarged, and troroughly revised; with 165 engravings, and four full-page plates. 800 pp. 556. Lea Brothers and Company, New York and Philadelphia, 1905.

A Treatise on Diagnostic Methods of Examination. By Prof. Dr. H. Sahli, of Bern. Edited, with additions, by Francis P. Kinnicutt, M.D., Professor of Clinical Medicine, Columbia University, N. Y.; and Nath'l Bowditch Potter, M.D., Visiting Physician to the City Hospital and to the French Hospital; and Consulting Physician to the Manhattan State Hospital, N. Y. Philadelphia and London: W. B. Saunders & Co., 1905. Octavo of 1008 pages, profusely illustrated. Cloth, $6.50 net; Half Morocco, $7.50 net.

A Treatise on Diseases of the Skin. For the use of advanced Students and Practitioners. By Henry W. Stelwagon, M.D., Ph. D., Professor of Dermatology, Jefferson Medical College, Philadelphia; and Clinical Professor of Dermatology, Woman's Medical College, Philadelphia. Fourth Edition, Revised. Handsome octavo of 1135 pages, with 258 text-illustrations, and 32 full-page lithographic and half-tone plates. Philadelphia and London: W. B. Saunders & Co., 1905. Cloth, $6.00 net; Sheep or Half Morocco, $7.00 net.

Transactions of the American Otological Society. Thirty-Eighth Annual meeting, held at Boston, Mass., May 9 and 10, 1905. Volume 9, part 1.' Dr. F. L. Jack, secretary, Published by the Society. Mercury Publishing Co., Printers, New Bedford, Mass., 1905.

Transactions of the American Laryngological Association. Twenty-seventh Annual meeting, held at Atlantic City, N. J., June 1, 2, and 3, 1905. Dr. James E. Newcomb, secretary. Published by the Association. New York: Rooney and Allen Printing Co., 1905.

Laboratory Manual of Physiology. By Frederick C. Busch B.S., M.D., Professor of Physiology, Medical Department, University of Buffalo. Illustrated. Small Octavo, pp. 206. New York: William Wood & Co., 1905. (Price, $1.25)

Transactions of the Medical Society of the State of New York for the year 1905 (ninty-ninth annual meeting). Frederic C. Curtis, M.D., secretary. Published by the Society. Brandon Printing Co., Fort Orange Press, Albany. 1905.

The National Standard Dispensatory. Containing the Natural History, Chemistry, Pharmacy, Actions and Uses of Medicines, including those recognised in the Pharmacopeias of the United States, Great Britain and Germany, with numerous references to other foreign pharmacopœias. In accordance with the United States Pharmacopeia, 8th decennial revision of 1905 by authorisation of the Convention. By Hobart Amory Hare, B.Sc., M.D., Professor of Theraputics in the Jefferson Medical College, Philadelphia, Member of the Committee of Revision of the U. S. P.; Charles Caspari, Jr., Ph.G., Phar.D., Professor of Pharmacy in the Maryland College of Pharmacy, Baltimore, Member of the Committee of Revision of the U. S. P.; and Henry H. Rusby, M.D., Professor of Botany and Materia Medica in the College of Pharmacy of the City of New York, Member of the Committee of Revision of the U. S. P. Imperial octavo, 1858 pages, 478 engravings. Cloth $7.25 net; leather, $8.00 net. Thumb index, $.50 extra. Lea Brothers & Co., Publishers, Philadelphia and New York. 1905.

A Textbook of the Practice of Medicine. By James M. Anders, M.D., Ph.D., LL.D., Professor of Medicine and of Clinical Medicine at the Medico Chirurgical College, Philadelphia. Seventh edition, revised and enlarged. Octavo of 1297 pages, fully illustrated. Philadelphia and London: W. B. Saunders & Co. 1905. (Cloth, $5.50 net; sheep or half-morocco, $6.50 net.

Saunder's Medical Hand-Atlases. Atlas and Epitome of Diseases of the Skin. By Professor Dr. Franz Mracek, of Vienna. Edited, with additions, by Henry W. Stelwagon, M.D., Professor of Dermatology, Jefferson Medical College, Philadelphia. Second edition, revised, enlarged, and entirely reset. With 77 colored lithographic plates, 50 half-tone illustrations, and 272 pages of text. Philadelphia and London: W. B. Saunders & Co. 1905. (Cloth. $4.00 net.)

A Manual of Diseases of the Nose and Throat. By Cornelius Godfrey Coakley, A.M., M.D., Professor of Laryngology in the University and Bellevue Hospital Medical College; Laryngologist to Columbus Hospital; Consulting laryngologict to the New York Board of Health, etc. etc. Third edition, revised and enlarged. Illustrated with 118 engravings and five colored plates, pp. 594. Lea Brothers & Co., New York and Philadelphia. 1905. (Price: $2.75)

Wilcox. Materia Medica and Pharmacy. In accordance with the new "U. S. Pharmacopœia," 1905. A Text-Book for Students of Medicine and Pharmacy, based upon the Fifth Edition of White & Wilcox's "Materia Medica and Theraputics." By Reynold Webb Wilcox. M.A., M.D., LL.D., Professor of Medicine at the New York Post Graduate Medical School and Attending Physician to the Hospital, etc. etc.; Vice-Chairman of the Revision Committe of the U. S. P. Octavo. (Cloth, $2.50 net.)

Physicians Pocket Account Book. A book for daily accounts of professional services. By J. J. Taylor, M.D. Published by The Medical Council, Philadelphia. 1905. (Price: $1.00)

A Manual of Chemistry. A Guide to Lectures and Laboratory Work for Beginners in Chemistry. A Text-Book specially adapted for Students of Pharmacy and Medicine. By W. Simon, Ph.D., M. D., Professor of Chemistry and Toxicology, College of Physicians and Surgeons, Baltimore; Professor of Chemistry in the Maryland College of Pharmacy. Eighth edition, thoroughly revised. In one 8vo. volume of 643 pages, with 66 engravings and 9 colored plates illustrating 64 of the most important chemical tests. New York 1905. Lea Brothers & Co., Philadelphia and New York. (Cloth, $3.00 net)

A Textbook of Diseases of Women. By Barton Cooke Hirst, M.D., Professor of Obstetrics, University of Pennsylvania. Second edition revised and enlarged. Octavo of 741 pages, with 701 original illustrations, many in colors. Philadelphia and London: W. B. Saunders & Co. 1905. (Cloth, $5.00 net.; sheep or half morocco, $6.00 net.)

Abdominal Operations. By B. G. A. Moynihan, M.S. (London), F.R.C.S., Senior Assistant Surgeon to Leeds General Infirmary, England. Octavo of 695 pages, with 250 original illustrations. Philadelphia and London: W. B. Saunders & Co. 1905. (Cloth $7.00 net)

